# Very long intergenic non-coding RNA transcripts and expression profiles are associated to specific childhood acute lymphoblastic leukemia subtypes

**DOI:** 10.1371/journal.pone.0207250

**Published:** 2018-11-15

**Authors:** Maxime Caron, Pascal St-Onge, Simon Drouin, Chantal Richer, Thomas Sontag, Stephan Busche, Guillaume Bourque, Tomi Pastinen, Daniel Sinnett

**Affiliations:** 1 CHU Sainte-Justine Research Center, Montreal, Quebec, Canada; 2 Department of Human Genetics, McGill University, Montreal, Quebec, Canada; 3 Department of Pediatrics, University of Montreal, Montreal, Quebec, Canada; German Cancer Research Center (DKFZ), GERMANY

## Abstract

Very long intergenic non-coding RNAs (vlincRNAs) are a novel class of long transcripts (~50 kb to 1 Mb) with cell type- or cancer-specific expression. We report the discovery and characterization of 256 vlincRNAs from a cohort of 64 primary childhood pre-B and pre-T acute lymphoblastic leukemia (cALL) samples, of which 61% are novel and specifically expressed in cALL. Validation was performed in 35 pre-B and pre-T cALL primary samples. We show that their expression is cALL immunophenotype and molecular subtype-specific and correlated with epigenetic modifications on their promoters, much like protein-coding genes. While the biological functions of these vlincRNAs are still unknown, our results suggest they could play a role in cALL etiology or progression.

## Introduction

Childhood acute lymphoblastic leukemia (cALL) represents approximately 25% of all pediatric cancer cases. Despite remarkable improvements in survival, with 5 year event-free survival rates of approximately 80%, non-responding or relapsing patients still represent one of the most frequent cause of disease-related death in children [1]. Childhood ALL is a complex disease comprising multiple molecular subtypes with distinctive somatic genetic alterations such as aneuploidy, chromosomal rearrangements, and point mutations [1]. High hyperdiploid cases (HHD) and those harboring the t(12;21) [*ETV6/RUNX1*] rearrangement, together representing about half of pre-B cALL cases. Both subtypes are associated with a good prognosis [1, 2]. Other less frequent (< 10%) subtypes, such as MLL-rearranged, t(1;19) [*TCF3/PBX1*], or t(9;22) [*BCR/ABL1*], are associated with intermediate to poor outcomes [1, 2]. These genetic alterations contribute to leukemogenesis by altering key regulatory processes, subverting normal proliferation control, blocking differentiation, and promoting resistance to death signals [2]. Although ~75% of cALL cases can be currently sub-classified clinically using standard cytology or molecular diagnostics techniques [1, 2], accurate patient risk stratification is still an ongoing challenge. Interestingly, a recent study showed that expression profiles could classify up to 98% of cases [3]. While these studies primarily focused on the analysis of protein-coding transcripts [3–6], long non-coding RNA (lncRNA) transcripts have also been shown to have pre-B cALL subtype-specific expression and can modulate cell proliferation, apoptosis, migration, and treatment resistance [4–7]. Recently, a new class of lncRNAs known as “very long intergenic non-coding RNAs” (vlincRNAs) has been described. So far only a few thousand vlincRNAs, whose size ranges from 50kb to 1Mb, have been identified. However, it is known that these transcripts show cell type-specific expression patterns and seem to have biological functions [8–10]. In this study, we described vlincRNA populations expressed in cALL primary samples through whole-transcriptome sequencing, assessed their cALL-subtype specificity, and investigated putative expression regulation mechanisms. The insight gained from our results on this new class of transcripts will spur further research on their expression and function not only in cALL but also in other cancer types.

## Results

### Identification of vlincRNAs expressed in cALL patients

**Fig 1:**
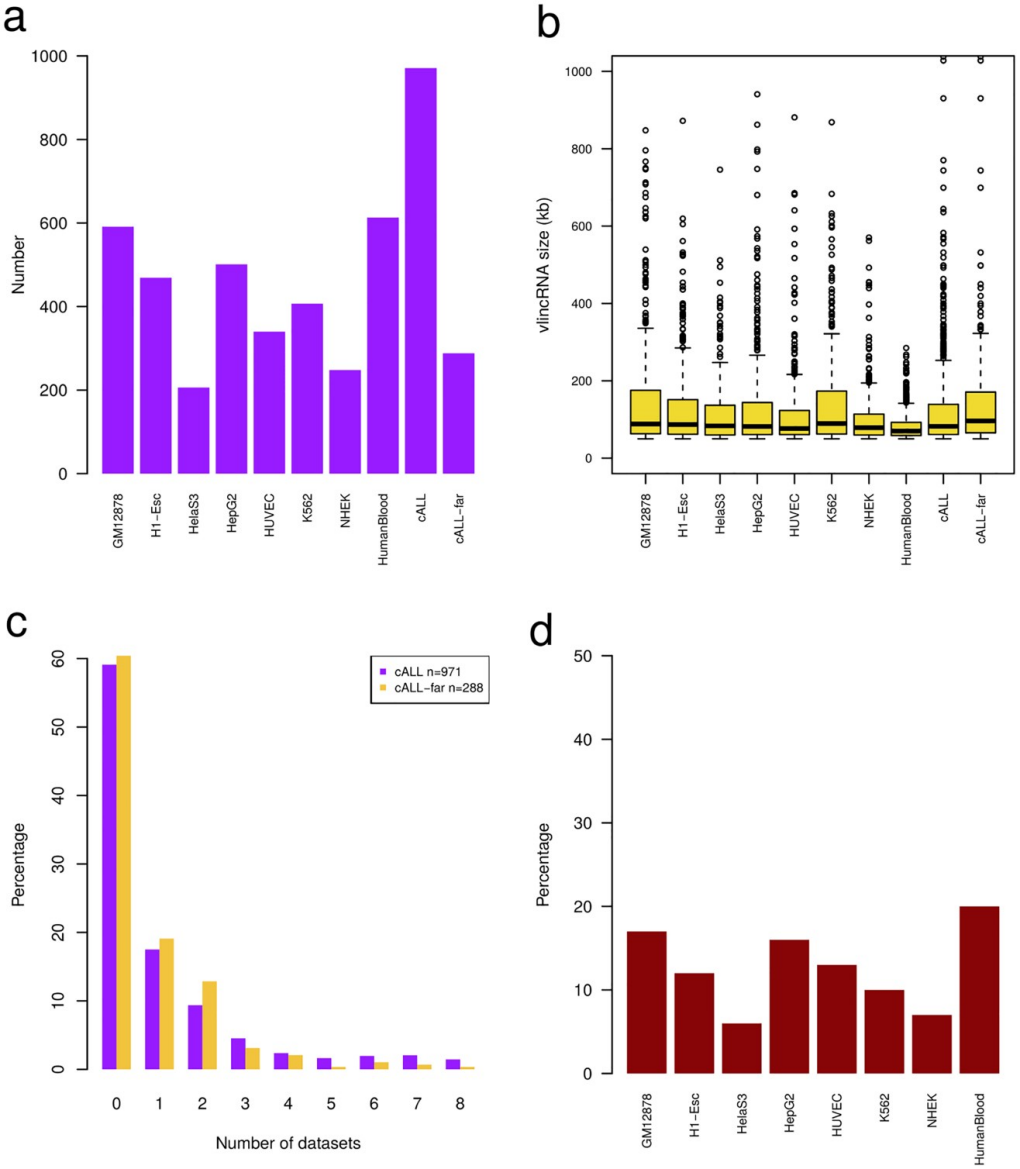
Characterization of cALL-derived vlincRNAs. (A) Number of vlincRNAs previously reported for diverse cell types (GM12878, H1-Esc, HelaS3, HepG2, HUVEC, K562, NHEK, HumanBlood) [9] and discovered in our cALL dataset, including ‘far-from-genes’ cALL vlincRNAs that are at least 10 kb away from protein-coding genes. (B) Boxplot of vlincRNA transcript sizes. (C) Percentage of all (n = 971) and ‘far-from-genes’ (n = 288) cALL vlincRNAs overlapping vlincRNAs a given number of public datasets. (D) Percentage of cALL vlincRNAs overlapping vlincRNAs from public datasets (HumanBlood data is unstranded).

We sequenced the whole transcriptome of 64 cALL patients (57 pre-B and 7 pre-T) and 4 matched normal cell populations derived from human cord blood (3 CD10^+^CD19^+^ pre-B and 1 CD3^+^ pre-T) (S1 Table; [6]). In this “discovery cohort” we identified a total of 971 expressed vlincRNAs with a median size of 82.2 kb [50 kb—1.03 Mb], in line with previous estimates and supporting the sensitivity of the discovery method (Fig 1A and 1B; [9]). When compared to available public datasets [8, 9], we found that 59% (574 / 971) of vlincRNAs were unique and specific to our cALL samples (< 25% reciprocal strand specific overlap) while the remaining 41% overlapped with those found in at least one existing dataset (Fig 1C and 1D). We identified a high-confidence subset of 288 vlincRNAs (31.4%; 288 / 917) located at least 10 kb away from protein coding genes on either strand. From these, we selected 256 autosomal vlincRNAs having at least 100 reads per sample in a minimum 3 samples to perform differential expression analyses.

### VlincRNA expression is pre-B cALL molecular subtype-specific

**Fig 2:**
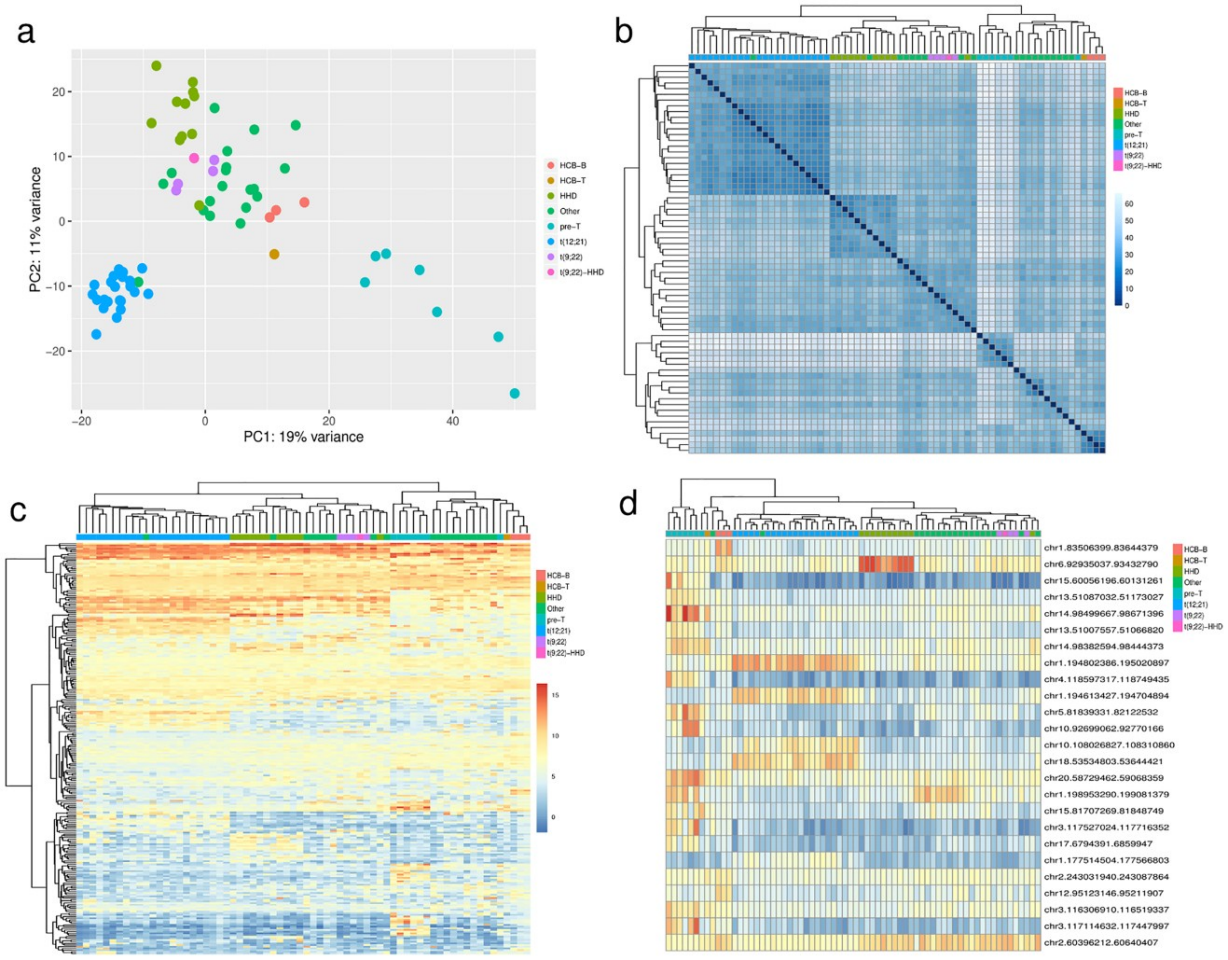
Subtype-specific classification of cALL using vlincRNA expression profiles. (A) PCA plot of the discovery samples (n = 68) using the DESeq2 regularized log transform (rld) normalized read counts of minimally expressed cALL vlincRNAs (n = 256). (B) Hierarchical clustering of discovery samples using Euclidean distance on vlincRNA normalized rld expression values. Cluster purity = 0.85 using 8 clusters. (C) Heatmap of vlincRNA normalized rld expression values in the discovery samples. (D) Heatmap of normalized rld expression values of the top 25 significantly differentially expressed cALL vlincRNAs (adj. p-value < 0.05) in the discovery samples using the likelihood ratio test on subtypes with DESeq2. HCB-B: CD19^+^CD10^+^ pre-B cells isolated from human cord blood. HCB-T: CD3^+^ pre-T cells isolated from human cord blood.

Since it was previously reported that lncRNA expression profiles can accurately classify pre-B cALL molecular subtypes [4, 7], we investigated whether this also held true for vlincRNAs. Principal component analysis on the selected 256 vlincRNAs showed cALL immunophenotype-specific expression patterns highlighted by pre-B and pre-T cALL cases clustering separately (Fig 2A). We observed cALL subtype discrimination between pre-T, HHD, t(12;21) [*ETV6*/*RUNX1*], t(9;22) [*BCR*/*ABL1*], and unclassified (“Other”) pre-B cALL subtypes when performing hierarchical clustering using vlincRNA normalized expression values (cluster purity = 0.85; Fig 2B). Furthermore, we observed subtype-specific vlincRNA expression patterns when either all 256 vlincRNAs (Fig 2C) or the top 25 vlincRNAs differentially expressed in primary cALL samples (relative to control) are considered (Fig 2D). Interestingly, a sample originally classified as “Other” at diagnosis clustered with t(12;21) samples using vlincRNA expression (Fig 2). We determined by RT-PCR that the t(12;21) [*ETV6*/*RUNX1*] fusion was indeed expressed and reassigned this sample to the t(12;21) [*ETV6*/*RUNX1*] subtype.

**Fig 3:**
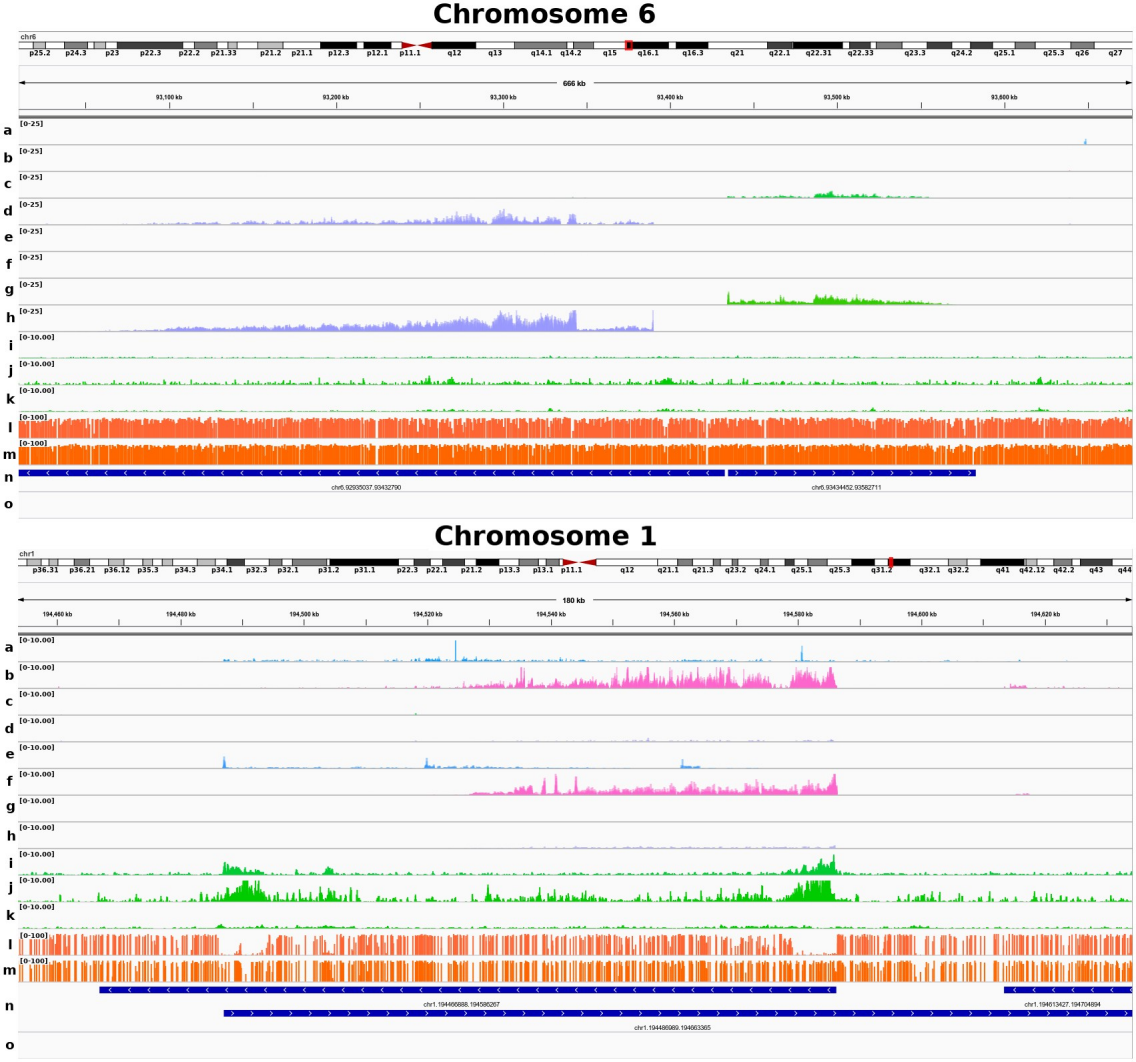
Genome wide tracks of gene expression, active histone marks and CpG methylation of subtype-specific cALL vlincRNAs. Epigenetic tracks of vlincRNAs expressed in specific subtypes. Top, HHD-specific vlincRNAs expressed on chromosome 6 in both the SOLiD and Illumina sequencing platforms. Bottom, t(12;21)-specific vlincRNAs expressed on chromosome 1 in both the SOLiD and Illumina sequencing platforms. Splicing patterns are more clearly defined with Illumina RNA-seq data. (A,B) Normalized read coverage of t(12;21) vlincRNA expression (SOLiD platform, + and—strands). (C,D) Normalized read coverage of HHD vlincRNA expression (SOLiD platform, + and—strands). (E,F) Normalized read coverage of t(12;21) vlincRNA expression (Illumina platform, + and—strands). (G,H) Normalized read coverage of HHD expression (Illumina, + and—strands). (I,J,K) Normalized read coverage of the H3K4me3, H3K27ac and H3K4me1 ChIP-seq histone marks in the t(12;21) pool sample. (L,M) WGBS methylation levels of the merged t(12;21) cases (n = 3) and the CD10^+^CD19^+^ control cells. (n) Coordinates of vlincRNA transcripts (n = 288) found in the discovery samples (n = 68). (o) RefSeq gene annotations.

Some vlincRNAs had expression levels orders of magnitude higher in specific subtypes, suggesting a subtype-specific role (e.g., HHD-specific or t(12;21)-specific vlincRNAs shown in Fig 3). To assess the robustness of these findings, we further analyzed the transcriptome of 35 independent primary samples (30 pre-B and 5 pre-T) as a replication cohort (S1 Table) using the Illumina HiSeq2500/4000 platforms (see Methods). PCA analysis and hierarchical clustering using minimally expressed vlincRNAs in the replication cohort (n = 273/288) confirmed the subtype-specific expression patterns observed in the discovery cohort (cluster purity of 0.94), S2A and S2B Fig). Subtype-specific vlincRNA expression between discovery and replication samples also showed strong Pearson correlations (S2C Fig), confirming that vlincRNA expression patterns are robust reliable discriminators of cALL subtypes. We also looked at the expression levels of t(12;21)-specific vlincRNAs in REH cells and found similar expression patterns in this cell line (S2D Fig). Interestingly, we observed different vlincRNA expression profiles for pre-B and pre-T cALLs, indicating that the expression of certain vlincRNA is cancer type-specific.

### Epigenetic regulation of cALL-specific vlincRNAs

**Fig 4:**
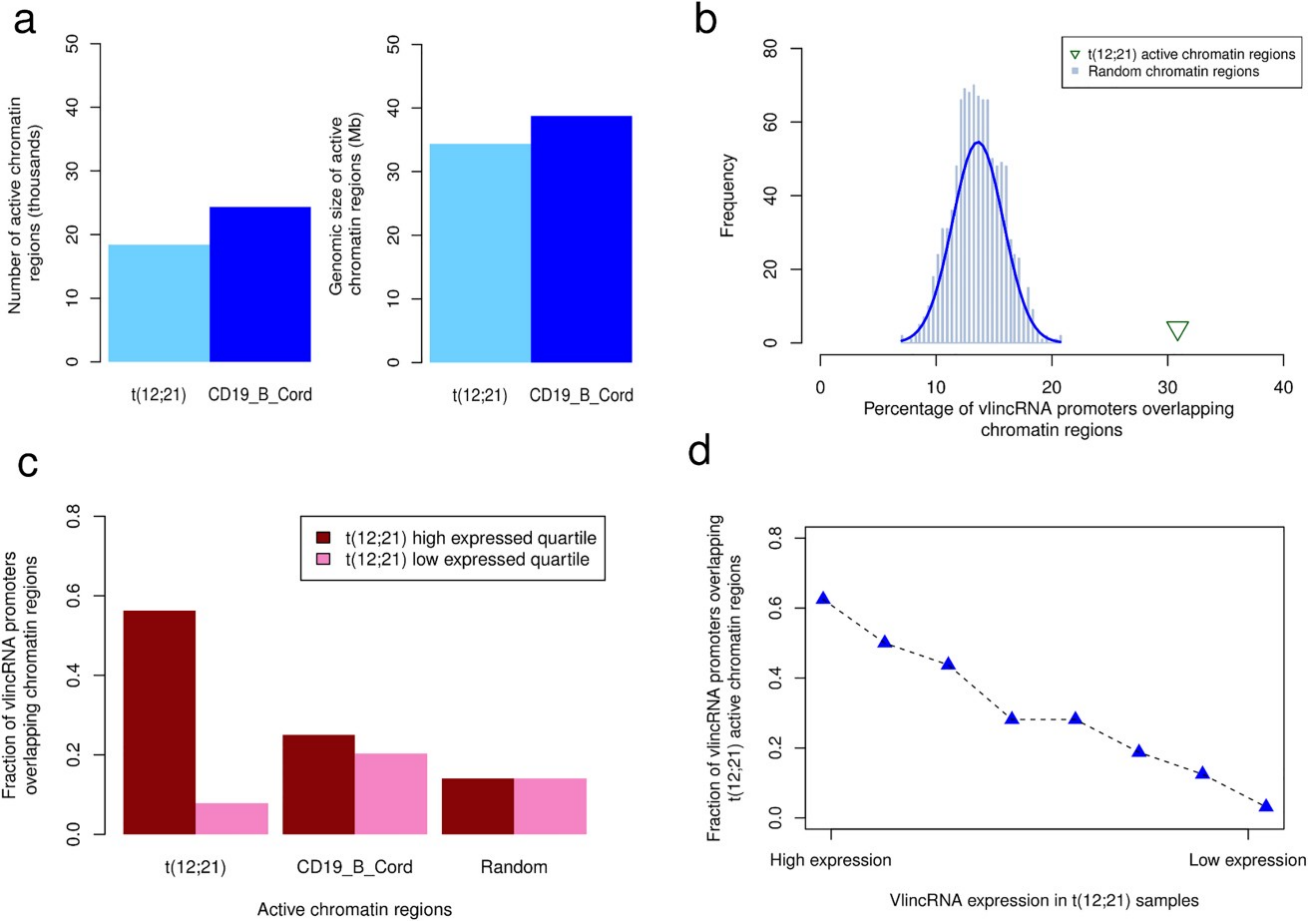
Epigenetic regulation at cALL vlincRNA promoters. (a) Number (left) and genomic size (right) of active chromatin regions in two t(12;21) samples and CD19^+^ B cord. Active chromatin regions in the t(12;21) samples were generated using ChromHMM on three chromatin marks (H3K4me3, H3K27ac and H3K4me1) and retaining states having the H3K4me3 mark present. Active CD19^+^ B cord chromatin regions were obtained from the Roadmap Epigenomics Project [14]by keeping states having the H3K4me3 mark present (states 1,2,3,10, and 11) from the 15-state model. (b) Percentage of vlincRNA promoters (n = 256) overlapping active t(12;21) chromatin regions (30.9%; 79 / 256) and random regions (mean 14.06%; 36 / 256). Random regions were generated a thousand times using shuffleBed from bedtools. (c) Percentage of vlincRNA promoters overlapping active t(12;21) chromatin regions, active CD19^+^ B cord chromatin regions and random regions in the t(12;21) high and low expressed vlincRNA quartiles (Q1 and Q4, n = 64). (d) Fraction of vlincRNA promoters overlapping active t(12;21) chromatin regions sorted by t(12;21) vlincRNA expression (8 bins of 32 promoters). HCB-B: CD19^+^CD10^+^ pre-B cells isolated from human cord blood.

**Fig 5:**
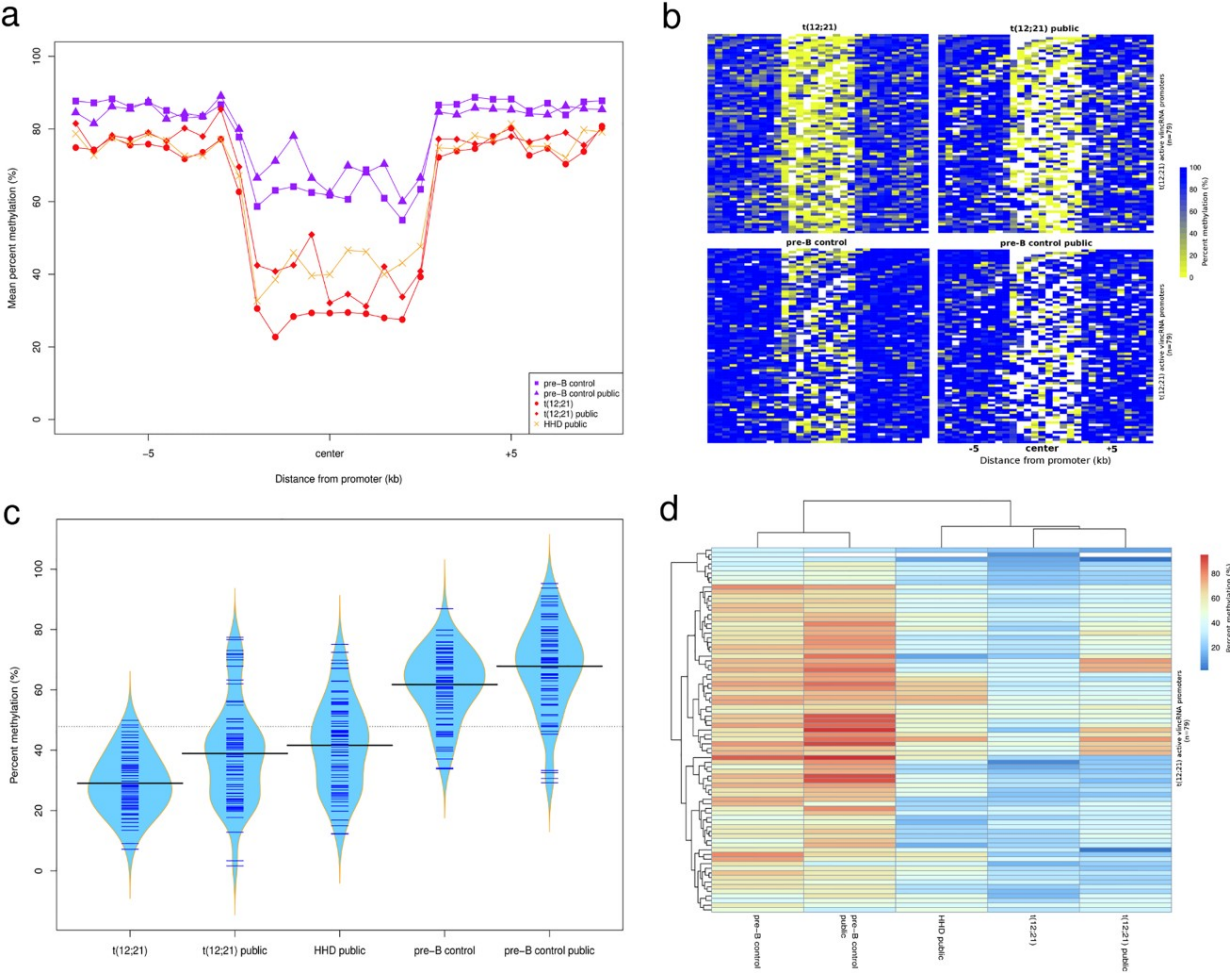
Methylation regulation of t(12;21) active vlincRNA promoters. (a) WGBS mean methylation levels of t(12;21) active promoters (n = 79) in t(12;21), HHD and pre-B control CD10^+^CD19^+^ datasets from this study and public data [11]. Each data point represents 10% of promoter size and flanking regions size (±10kb). (b) WGBS methylation heatmap of t(12;21) active promoters in t(12;21) and pre-B control CD10^+^CD19^+^ datasets from this study and public data (white = no covered CpGs). Each column represents 10% of promoter size and flanking regions size (±10kb). (c) DNA methylation beanplot of t(12;21) active promoters in t(12;21), HHD and pre-B control CD10^+^CD19^+^ datasets from this study and public data. (d) Hierarchical clustering of methylation levels of t(12;21) active promoters in t(12;21), HHD and pre-B control CD10^+^CD19^+^ datasets showing similar methylation profiles between this study and public data.

To delineate the basis of the vlincRNA expression regulation in cALL, we investigated epigenetic regulation by generating ChIP-seq data of the H3K4me1, H3K4me3, and H3K27ac active chromatin marks from a pool of two additional t(12;21)[*ETV6*/*RUNX1*] cases. Nearly a third (30.9%; 79 / 256) of cALL-specific putative vlincRNA promoters (±10 kb) overlapped active chromatin regions, representing a significant enrichment (2.2-fold enrichment; Fisher p-value = 4.0x10^-6^). Enriched active chromatin region overlaps was positively correlated with vlincRNA expression in t(12;21) [*ETV6*/*RUNX1*] samples: the highest expression quartile has a higher fraction of overlap than the lowest quartile (Q1: 56.3% vs. Q4: 7.8%; Fig 4). DNA methylation was also investigated to further characterize the epigenetic landscape of cALL vlincRNAs by performing whole genome bisulfite sequencing (WGBS) of three t(12;21)[*ETV6*/*RUNX1*] cases and one control CD10^+^CD19^+^ pre-B cell sample isolated from human cord blood. This experiment showed that the 79 active vlincRNA promoters described above were hypomethylated compared to control cells. This observation is further supported by the analysis of publicly available WGBS results [11] and 450K DNA methylation data [12, 13] of 242 samples (see Methods; Fig 5 and S4 Fig). Transcription factor motif enrichment analyses of highest expressed quartiles vlincRNA candidate promoters (±10kb; n = 64) in the t(12;21) and HHD subtypes revealed distinct regulators in both subtypes (S5 Fig). Motifs from the *ETS* transcription factor family, with roles in tissue development and cancer progression, were significantly enriched in the t(12;21) subtype, while binding motifs for AP-1 subunits *JUN* and *FOS*, implicated in apoptosis, proliferation, and differentiation, were significantly enriched in the HHD subtype.

**Fig 6:**
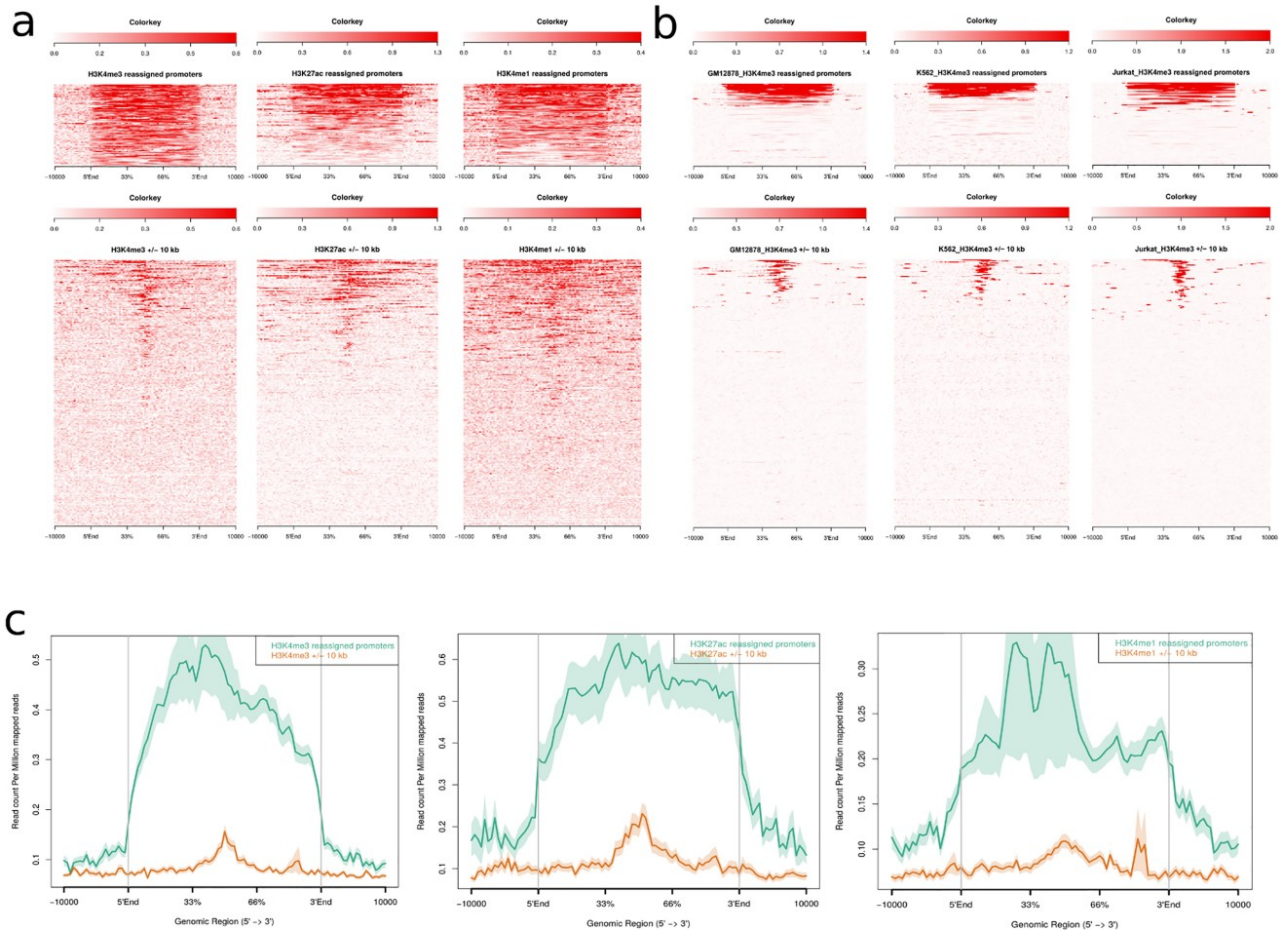
Histone mark ChIP-seq read density plots of candidate t(12;21) active promoters. (a) Heatmaps of H3K4me3, H3K27ac and H3K4me1 ChIP-seq normalized read densities for candidate (bottom, ± 10kb around the 5’ start of vlincRNAs, n = 256) and reassigned (top, n = 79) t(12;21) active vlincRNA promoters. (b) H3K4me3 ChIP-seq normalized read densities in GM12878, K562 and Jurkat cell lines on candidate and reassigned t(12;21) active vlincRNA promoters. (c) ChIP-seq normalized read density plots of the H3K4me3, H3K27ac and H3K4me1 histone marks for candidate and reassigned t(12;21) vlincRNA promoters.

Finally, we investigated whether other hematological cell types exhibited a similar trend by looking at H3K4me3 read density on vlincRNA promoters overlapping active chromatin regions (identified above) in three ENCODE cell lines: GM12878 (B-lymphocytes), K562 (chronic myeloid leukemia), and Jurkat (pre-T ALL). Interestingly, these cell lines also showed active chromatin enrichment on a fraction of vlincRNA promoters identified in t(12;21) [*ETV6*/*RUNX1*] samples, suggesting that vlincRNA expression could be regulated through epigenetic mechanisms (Fig 6; [15]). Given that ~40% of vlincRNAs expressed in cALL are not cALL-specific, it is not surprising that a subset of t(12;21) [*ETV6*/*RUNX1*] actively regulated vlincRNAs could be under H3K4me3 activation in other hematological cell lines.

## Discussion

In this study, we identified and characterized very long intergenic non-coding RNAs (vlincRNA) expression patterns in 64 primary cALL samples (57 pre-B and 7 pre-T) and showed that they are specific to cALL subtypes and that epigenetic modifications correlated with their expression. We found 971 vlincRNA transcripts expressed in cALL primary samples, of which 59% (574 / 971) were unique to our dataset. We further selected 256 high-confidence autosomal vlincRNAs having at least 100 reads per sample in a minimum 3 samples to perform differential expression analyses (156 / 256, or 61% unique to cALL). The median size and range of the vlincRNA transcripts we identified was of 82.2 kb [50 kb—1.03 Mb], which is concordant with previously reported sizes (~50 kb—1 Mb), with a median size of ~80 kb [9, 16]. Although it was reported that vlincRNAs are mostly unspliced [10], we have observed putative splicing patterns in some vlincRNAs (Fig 3), indicating that they could be expressed in both spliced and unspliced forms or less likely be novel pre-mRNAs.

Despite the current cure rate, cALL patients whose disease is refractory to treatment or relapses face a dismal prognosis. In addition, this high cure rate has been achieved through risk-based treatments administered at the expense of considerable toxicity and decreased quality of life. Indeed, more than 70% of young adult survivors will suffer from long-term effects because of their treatments [17, 18]. Thus, the development of improved risk stratification strategies leading to personalized and targeted treatment is thus essential to improve patient outcome and long-term quality of life. Previous studies done using microarray technology [5] or whole-transcriptome sequencing had shown that protein-coding [4, 7] or long non-coding RNA transcription profiles [4–7] could be used to discriminate cALL disease subtypes. Here, we have demonstrated that vlincRNA expression profiles can discriminate between cALL subtypes, particularly the t(12;21) [*ETV6*/*RUNX1*] and HHD subtypes, which together account for ~60% of pre-B cALL cases. These results, validated in 35 other primary cALL samples suggest that vlincRNA expression patterns can be used as molecular biomarkers for more accurate disease subtype classification. It would be interesting to see if these results scale with increased cohort size to reach the high cALL subtype classification accuracy demonstrated by Lilljebjorn *et al*. [3]. Furthermore, we show that vlincRNA expression is cancer-specific as pre-B and pre-T cALL exhibit different expression patterns. This observation is concordant with that of St-Laurent and colleagues [9] which have reported a wide variation in vlincRNA expression pattern across cancerous and normal cell types. Although these are both acute leukemias, they originate from distinct cell types, play different role, carry specific molecular alterations and thus can be considered as different cancers. These data and the consistency in expression patterns observed across both our discovery and validation cohorts strongly suggest that they are not transcriptional noise but rather disease-specific and even disease subtype-specific.

We further showed that putative vlincRNA promoters are enriched in active chromatin histone marks and had lower DNA methylation levels than healthy cell counterparts. These data support St-Laurent *et al*.’s hypothesis of vlincRNA transcriptional epigenetic regulation [9]. Together these data strongly suggest that subtype-specific vlincRNA expression is regulated by epigenetic changes.

We demonstrated that vlincRNA expression is cALL subtype-specific, but the question remains about the function of these molecules in normal cells and their role in cancer etiology and progression. Silencing experiments performed on vlincRNAs expressed in K562 chronic myeloid leukemia cells resulted in an increase in apoptosis, particularly for transcripts that were more broadly expressed across cell types, suggesting that cell type-specific vlincRNAs have more specialized roles [9]. Others have shown that vlincRNAs are involved in senescence control [10]. Although further experiments would be required to confirm this, we speculate that cALL subtype-specific vlincRNAs could have distinct biological roles between subtypes. Other cALL vlincRNAs that are more broadly expressed across cALL subtypes or other cancer cell types could also play a role in proliferation or apoptosis; again, additional work is required to assess their roles.

In conclusion, we have identified cALL subtype-specific vlincRNAs transcripts whose expression is controlled by well-known epigenetic mechanisms and modulators. In addition to protein-coding genes and long non-coding RNAs, we have shown that vlincRNA expression profiles can accurately classify cALL subtypes.

## Materials and methods

### Sample cohort

Study samples consist of 102 cALL patients (90 pre-B and 12 pre-T, of which 57 pre-B and 7 pre-T belong to the RNA-seq “discovery cohort”) from the established Quebec Childhood ALL cohort [19] (S1 Table) and 4 control samples (3 pre-B CD10^+^CD19^+^ and 1 pre-T CD3^+^) isolated from human cord blood. Patients (50 females and 52 males) aged from 1–17 years (median 5.54 years) were diagnosed in the Division of Hematology-Oncology at the Sainte-Justine UHC, Montreal, Canada, between 1994 and 2017. They underwent treatment with Dana Farber Cancer Institute (DFCI) ALL Consortium protocols DFCI 91–01, 95–01, 2000–01, 2005–01, or 2011–01 [20]. Mononuclear cells were isolated from bone marrow (BM) aspirates or peripheral blood cells at diagnosis and contained a mean level of 90% leukemic blasts. The Sainte-Justine Institutional Review Board approved the research protocol, and written informed consent was obtained from all participating individuals or their parents.

### RNA sequencing

Total RNA was extracted from white blood cell pellets obtained from bone marrow or peripheral blood tissue, followed by a DNase treatment to remove possible genomic DNA contamination. The discovery and control samples were sequenced on the the SOLiD System (Life Technologies) following standard manufacturer protocols. Briefly, ribosomal RNAs were removed using the RiboMinus Eukaryote kit (Invitrogen), cDNA libraries for all samples were prepared using the SOLiD total RNA-seq kit (Applied Biosystems), RNA was fragmented into 100-200bp fragments, purified and ligated to SOLiD adapters, fragments were then reverse-transcribed and size-selected, cDNAs were enriched by PCR and purified, Clonally amplified beads were enriched and subjected to paired-end whole transcriptome sequencing (50bp x 35bp or 75bp x 35bp). Reads were aligned to the human genome (hg19) using the Lifescope Genomic Analysis Software (Applied Biosystems). Replication samples and the Reh cell line were sequenced on the Illumina platform). Briefly, ribosomal RNAs were removed using the Ribo-Zero human/mouse/rat included in the TruSeq Stranded Total RNA LT sample preparation kit (Illumina Cat#RS-122-2201) as per manufacturer’s protocol. Final libraries were quality controlled on a Bioanalyzer and underwent 75 or 100bp paired-end sequencing on Illumina HiSeq2500/4000 systems. Reads were aligned to the human genome (hg19) using STAR v2.4.2 [21]. All sequencing experiments were done at the Integrated Centre for Pediatric Clinical Genomics, Sainte-Justine UHC, Montreal, Canada. Whole transcriptome datasets are available on the Gene Expression Omnibus (GEO) under accession number GSE89071.

### VlincRNA identification and expression profiling

The vlincRNA identification procedure was derived from St-Laurent *et al*. [9]. Briefly, 68 SOLiD strand-specific total RNA-seq datasets (64 samples and 4 controls) were pooled, split by chromosome and filtered for mapping quality > = 40 using Picard (Broad Institute) and SAMtools [22]. Strand-specific base coverage was obtained using BEDTools’ genomeCoverageBed v2.25.0 [23]. Covered bases overlapping UCSC or RefSeq protein coding genes from the UCSC Table Browser (April 2016) were removed. Bases having less than Q3 (75%) read density or overlapping blacklisted regions (~170Mb total) were removed. Blacklisted regions consisted of ENCODE’s EncodeDacMapabilityConsensus [15], svelter’s exclude file [24] and canvas’ filter file [25]. Remaining covered bases were merged if less than 500bp apart and the resulting segments were merged if less than 10kb apart. Finally, strand-specific vlincRNAs were defined as segments longer than 50kb (n = 971). A subset of these, ’far from genes’ (n = 288), were defined as being at least 10 kb away from protein coding genes regardless of strand orientation. Expression read counts for the SOLiD datasets (n = 68) and the Illumina datasets (n = 35) were obtained using htseq-count v0.6.1p1 [26]. Autosomal vlincRNAs having at least 100 reads per sample in minimum 3 samples (n = 256) were used to perform differential expression analyses on the SOLiD dataset using the DESeq2 likelihood ratio test [27] on subtypes, with sequencing batch and one sva variable as covariates (Fig 2A and 2B) [28]. Expression cluster purity was calculated on eight clusters for the discovery cohort and five clusters for the replication cohort (cluster numbers returned by mclust [29]).

### Chromatin immunoprecipitation (ChIP) sequencing

ChIP-sequencing was performed on bone marrow aspirates or peripheral blood mononuclear cells of a pool of two t(12;21) cases using the previously described procedure [30]. Briefly, the DNA of 10 to 30 million cells was immunoprecipitated using the following antibodies: H3K4me1 (Abcam; ab8895), H3K4me3 (Diagenode; pAb-003-050), and H3K27ac (Abcam; ab4729). Library preparation for ChIP-seq assays was carried out using the Paired-End DNA Sample Prep Kit V1 (Illumina; PE-102-1001) and sequenced using the HiSeq2000 sequencing system (2 x 100bp) at the McGill University and Genome Quebec Innovation Center. Reads were aligned with bwa v.0.6.1 [31] and peaks were called using macs2 [32] with an input control and the “*—broad*” option.

### VlincRNA promoter epigenetic analyses

Candidate promoters of vlincRNAs were defined as ±10kb around the start of expression. Promoter coordinates were refined in the t(12;21) [*ETV6*/*RUNX1*] subtype by taking the corresponding ChIP-seq peaks of the H3K4me3, H3K4me1, and H3K27ac chromatin marks and using them as input in ChromHMM v.1.11 [33] to distribute chromatin into 8 states. Active promoter regions were defined as having the H3K4me3 mark present (n = 19313, total size = 35.9Mb, median size = 1.4kb; Fig 4A). Regions separated by less than 1kb were merged and those longer or equal to 400bp were retained. The longest active regions overlapping candidate promoter regions were retained and reassigned as being active promoters (Figs S3 and 6A and 6C). Active CD19^+^ B cord chromatin regions were obtained from the Roadmap Epigenomics Project [14] by keeping states having the H3K4me3 mark present (states 1,2,3,10, and 11) from the 15-state model. The background distribution of active overlapping regions was obtained by generating a thousand random genomic intervals based on t(12;21) [*ETV6*/*RUNX1*] putative promoter-active region sizes (Fig 4B). The relationship between expression and overlap of active promoters was obtained by grouping vlincRNAs into t(12;21) [*ETV6*/*RUNX1*] expression quartiles and reporting the percentage of overlap. Read density plots and heatmaps of chromatin marks around putative and active vlincRNA promoters were obtained using ngsplot v2.47 [33]. Motif analyses were done with HOMER [34] using the putative promoters (±10kb around the start of transcription) of the high expressed vlincRNA quartiles in the t(12;21) [*ETV6*/*RUNX1*] and HHD subtypes.

### Whole genome bisulfite sequencing (WGBS)

WGBS was performed on bone marrow aspirates or peripheral blood mononuclear cells of three t(12;21) [*ETV6*/*RUNX1*] cases and on one human cord blood CD10^+^CD19^+^ control using a previously described procedure [34]. Briefly, WGBS gDNA library preparations were carried out using the TruSeq DNA Sample Prep Kit v2 (Illumina) with an added bisulfite conversion step. Amplified libraries were validated and quantified on Bioanalyzer High Sensitivity DNA Chips and underwent 100bp paired-end sequencing on the Illumina HiSeq2000 system at the McGill University and Genome Quebec Innovation Center. Reads were aligned to a bisulfite-converted reference genome using bwa and methylation calls were obtained using nxtgen-utils [35]. Mean coverage of the three t(12;21) cases were 6X, 13X, and 20X and mean coverage of the pre-B CD10^+^CD19^+^ control was 18X (bisulfite conversion rates > 99.5%). The C/T read counts of the three t(12;21) cases were merged to create a single 39X high coverage dataset.

### 450K methylation arrays

IDAT files measuring DNA methylation at single CpG-site resolution from the Illumina Infinium HumanMethylation450 BeadChips Array were generated in-house and obtained from previous studies [12, 13]. Samples are four healthy B cell stages: mpp (n = 5), preB-I (n = 6), preB-II (n = 7) and immature B (n = 5) and three cALL subtypes: t(12;21) (n = 61), HHD (n = 82) and ’Other’ (n = 76). IDAT Files were processed using minfi [36] with funnorm normalization [37]. Failed probes (detection p-value > 0.01 in more than 20% of samples), probes on sex chromosomes and probes overlapping SNPs were removed. Beta values were used as estimates of methylation levels.

### Methylation analyses

WGBS methylation values upstream (-10kb), downstream (+10kb) and on active t(12;21) [*ETV6*/*RUNX1*] promoters were obtained by splitting the regions into ten equal-size bins. Methylation values of covered CpGs overlapping the bins were averaged. Boxplots and hierarchical clustering of promoter methylation levels were obtained by averaging methylation values of covered CpGs across the promoters. For 450K datasets, beta values of probes overlapping active t(12;21) [*ETV6*/*RUNX1*] promoters (34 promoters, 161 probes) were averaged to obtain one methylation value per promoter per sample.

### Data access

Whole transcriptome, ChIP-seq and WGBS datasets are available on the Gene Expression Omnibus (GEO) under accession numbers GSE89071 and GSE120677.

## Supporting information

S1 Fig Discovery of cALL vlincRNAs. Genome wide tracks illustrating the discovery procedure of vlincRNAs in cALL samples. Blue = forward strand, pink = reverse strand. (a,b) Strand specific RNA-seq read coverage of pooled discovery samples (n = 68, SOLiD platform). (c) RefSeq gene annotations. (d,e) Strand specific protein coding gene coordinates of merged UCSC and RefSeq annotations. (f,g) Strand specific bases covered by at least one read using genomeCoverageBed from bedtools. (h,i) Strand specific covered bases having more than 75% read density and not overlapping protein coding genes or blacklisted regions. (j) Strand specific transcripts longer than or equal to 50 kb after merging covered bases less than 500 bp apart and merging resulting segments less than 10 kb apart.
(TIF)

S2 Fig Subtype-specific classification of replication cALL samples using vlincRNA expression profiles. (a) PCA plot of the replication samples (Illumina platform, n = 35) using the DESeq2 regularized log transform (rld) normalized expression of minimally expressed cALL vlincRNAs (n = 273). (b) Hierarchical clustering of the replication samples using Euclidean distance on vlincRNA normalized rld expression values. Cluster purity = 0.94 using 5 clusters. (c) Subtype-specific Pearson correlations of vlincRNA mean log_2_ normalized expression across discovery and replication samples (the t(9;22)-HHD sample was not included in this analysis). (d) Normalized rld expression of the top five t(12;21)-specific vlincRNAs in REH cells. t(12;21)-specific vlincRNAs were determined as having a minimum of 2 fold change higher expression than in the other subtypes and sorting the fold change in descending order.
(TIF)

S3 Fig Redefining coordinates of t(12;21) active promoters using histone mark ChIP-seq data. (a,b) Overlay of strand specific RNA-seq normalized read coverage of the t(12;21) Illumina samples (n = 22, + and—strands respectively). (c,d,e) Normalized read coverage of the H3K4me3, H3K27ac and H3K4me1 ChIP-seq histone marks of the t(12;21) pool sample. (f,g) WGBS methylation levels of the merged t(12;21) cases (n = 3) and the CD10^+^CD19^+^ control sample respectively. (h) VlincRNA transcripts (n = 256) discovered in the cALL discovery samples (n = 68). (i) RefSeq gene annotations. (j) Active chromatin regions containing the H3K4me3 mark from ChromHMM. (k) Candidate promoter regions ±10 kb around the 5’ start of vlincRNAs. (l) Redefined promoter coordinates by keeping the largest active chromatin regions overlapping the candidate promoters. A third (30.9%; 79 / 256) of the candidate vlincRNA promoters are defined as active.
(TIF)

S4 Fig Pearson correlations of 450K methylation levels of t(12;21) active promoters in four normal B cell stages and three cALL subtypes. Pairwise Pearson correlations of 450K methylation levels of t(12;21) active promoters (n = 34) in four healthy B cell stages (mpp, preB-I, preB-II, immature B) and three cALL subtypes (t(12;21), HHD, ’Other’) from both in-house and public datasets (242 samples total). 450K beta values were used as methylation levels. For each promoter, methylation levels were obtained by averaging the values of all overlapping 450K probes. For more than half of t(12;21) active promoters (57%; 45 / 79), no probes overlapped. Circle size increases as Pearson correlation decreases.
(TIF)

S5 Fig Transcription factor motif enrichment in candidate promoters of highly expressed t(12;21) and HHD vlincRNAs. Significantly enriched motifs (q-value < 0.05) in candidate promoters (±10 kb around the 5’ start of vlincRNAs) of t(12;21) and HHD high expressed quartiles vlincRNAs (Q4, n = 64).
(TIF)

S1 Table Study samples. (XLSX)

S2 Table List of vlincRNAs discovered in the cALL samples. (XLSX)
