# Supplementary figures and images for: Very long intergenic non-coding RNA transcripts and expression profiles are associated to specific childhood acute lymphoblastic leukemia subtypes
Source: PLoS One. 2018 Nov 15;13(11):e0207250. doi: 10.1371/journal.pone.0207250 (PMC6237371; doi:10.1371/journal.pone.0207250)

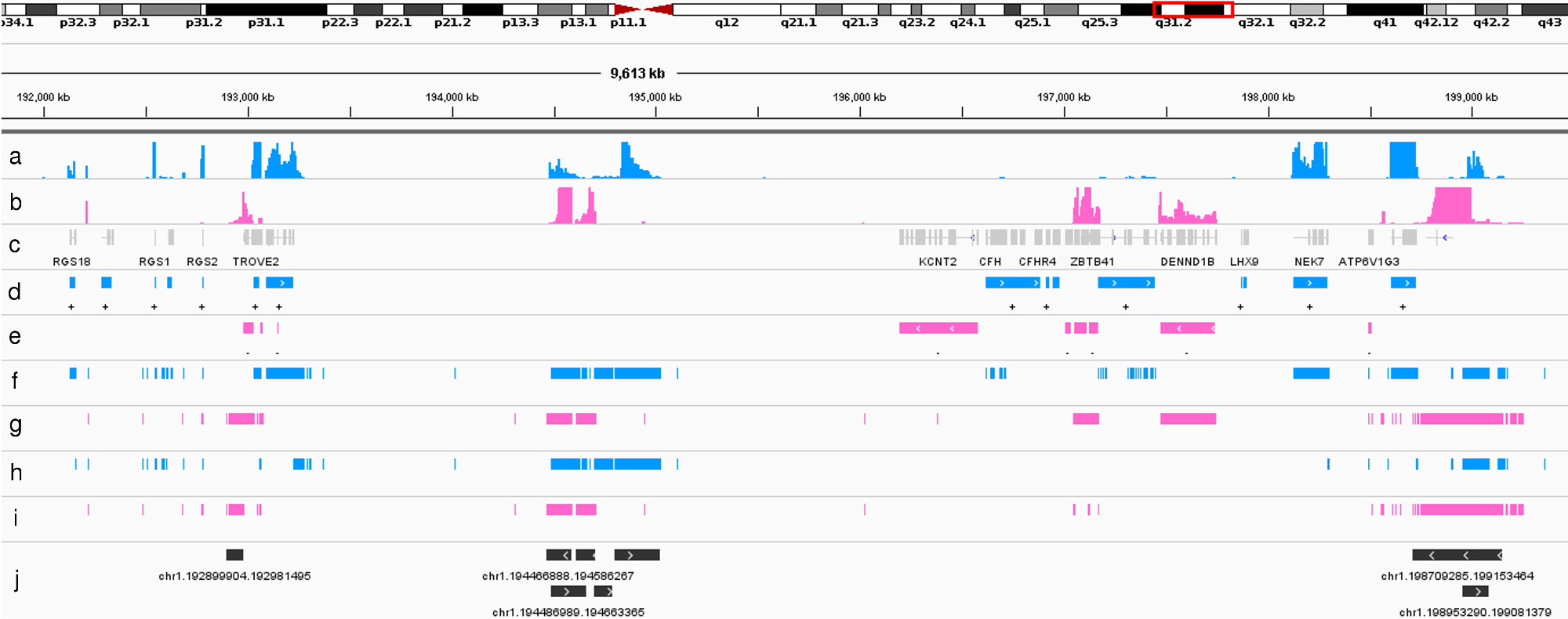

Supplement: S1 Fig — Genome wide tracks illustrating the discovery procedure of vlincRNAs in cALL samples. Blue = forward strand, pink = reverse strand. (a,b) Strand specific RNA-seq read coverage of pooled discovery samples (n = 68, SOLiD platform). (c) RefSeq gene annotations. (d,e) Strand specific protein coding gene coordinates of merged UCSC and RefSeq annotations. (f,g) Strand specific bases covered by at least one read using genomeCoverageBed from bedtools. (h,i) Strand specific covered bases having more than 75% read density and not overlapping protein coding genes or blacklisted regions. (j) Strand specific transcripts longer than or equal to 50 kb after merging covered bases less than 500 bp apart and merging resulting segments less than 10 kb apart. (TIF) [file pone.0207250.s001.tif]

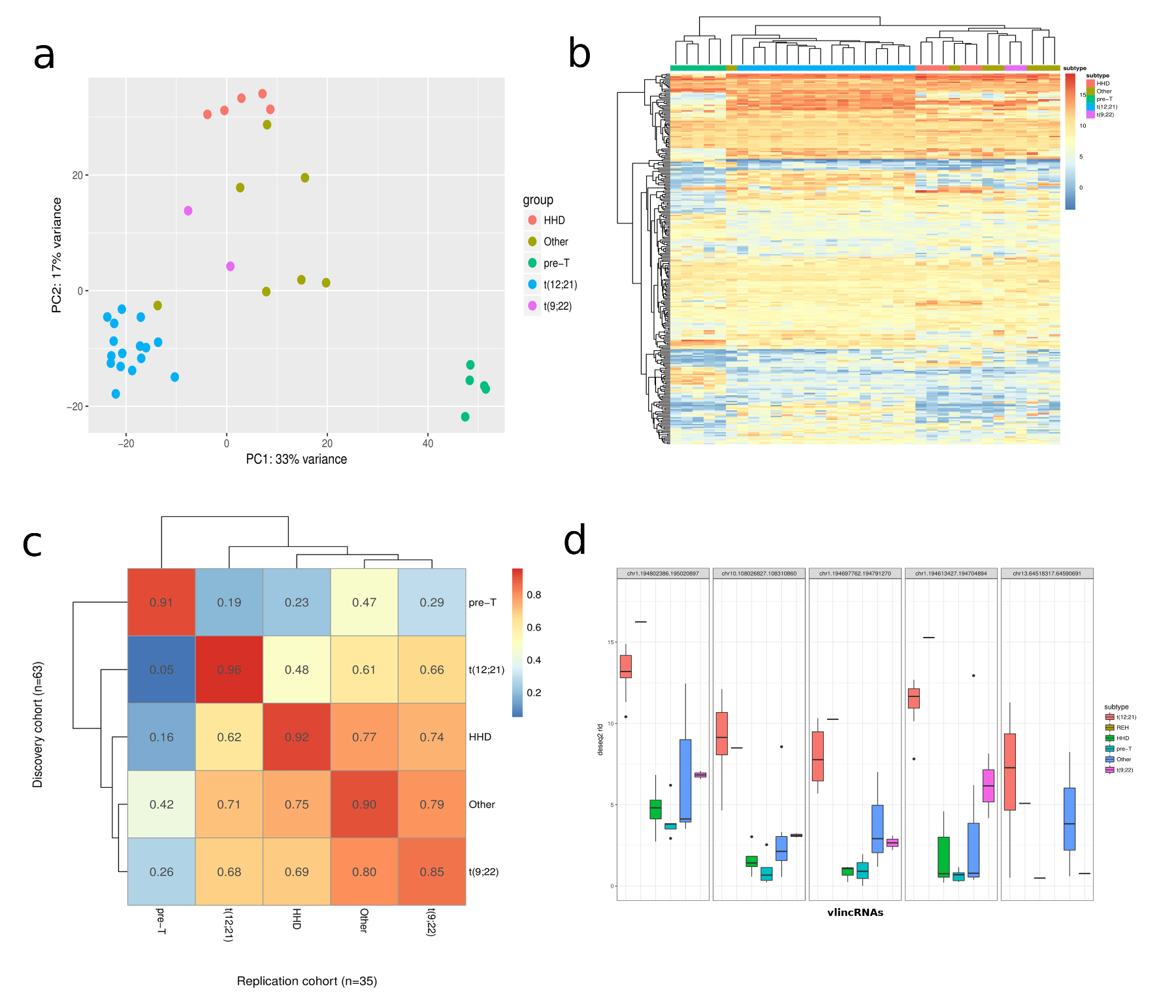

Supplement: S2 Fig — (a) PCA plot of the replication samples (Illumina platform, n = 35) using the DESeq2 regularized log transform (rld) normalized expression of minimally expressed cALL vlincRNAs (n = 273). (b) Hierarchical clustering of the replication samples using Euclidean distance on vlincRNA normalized rld expression values. Cluster purity = 0.94 using 5 clusters. (c) Subtype-specific Pearson correlations of vlincRNA mean log2 normalized expression across discovery and replication samples (the t(9;22)-HHD sample was not included in this analysis). (d) Normalized rld expression of the top five t(12;21)-specific vlincRNAs in REH cells. t(12;21)-specific vlincRNAs were determined as having a minimum of 2 fold change higher expression than in the other subtypes and sorting the fold change in descending order. (TIF) [file pone.0207250.s002.tif]

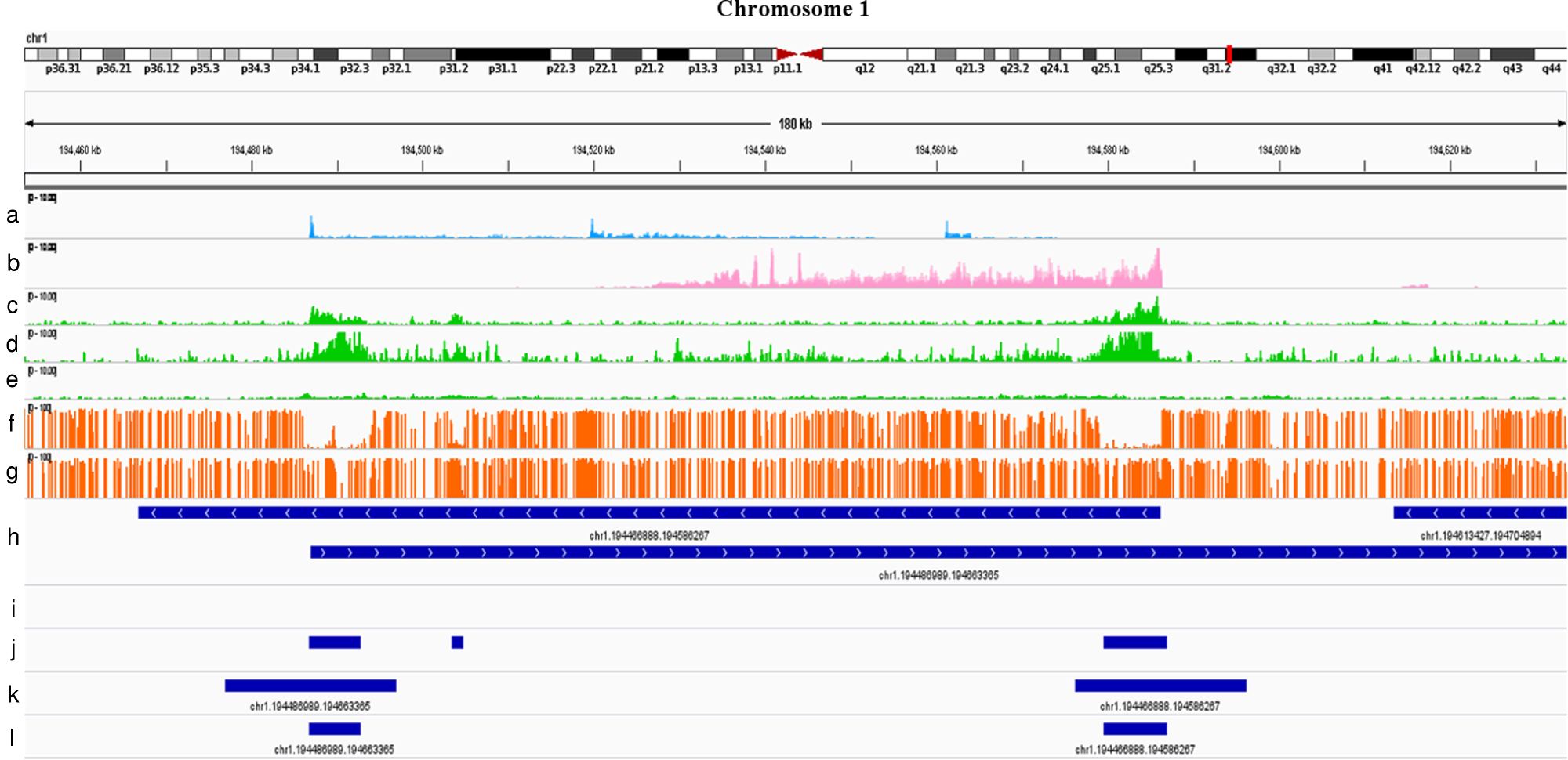

Supplement: S3 Fig — (a,b) Overlay of strand specific RNA-seq normalized read coverage of the t(12;21) Illumina samples (n = 22, + and—strands respectively). (c,d,e) Normalized read coverage of the H3K4me3, H3K27ac and H3K4me1 ChIP-seq histone marks of the t(12;21) pool sample. (f,g) WGBS methylation levels of the merged t(12;21) cases (n = 3) and the CD10+CD19+ control sample respectively. (h) VlincRNA transcripts (n = 256) discovered in the cALL discovery samples (n = 68). (i) RefSeq gene annotations. (j) Active chromatin regions containing the H3K4me3 mark from ChromHMM. (k) Candidate promoter regions ±10 kb around the 5’ start of vlincRNAs. (l) Redefined promoter coordinates by keeping the largest active chromatin regions overlapping the candidate promoters. A third (30.9%; 79 / 256) of the candidate vlincRNA promoters are defined as active. (TIF) [file pone.0207250.s003.tif]

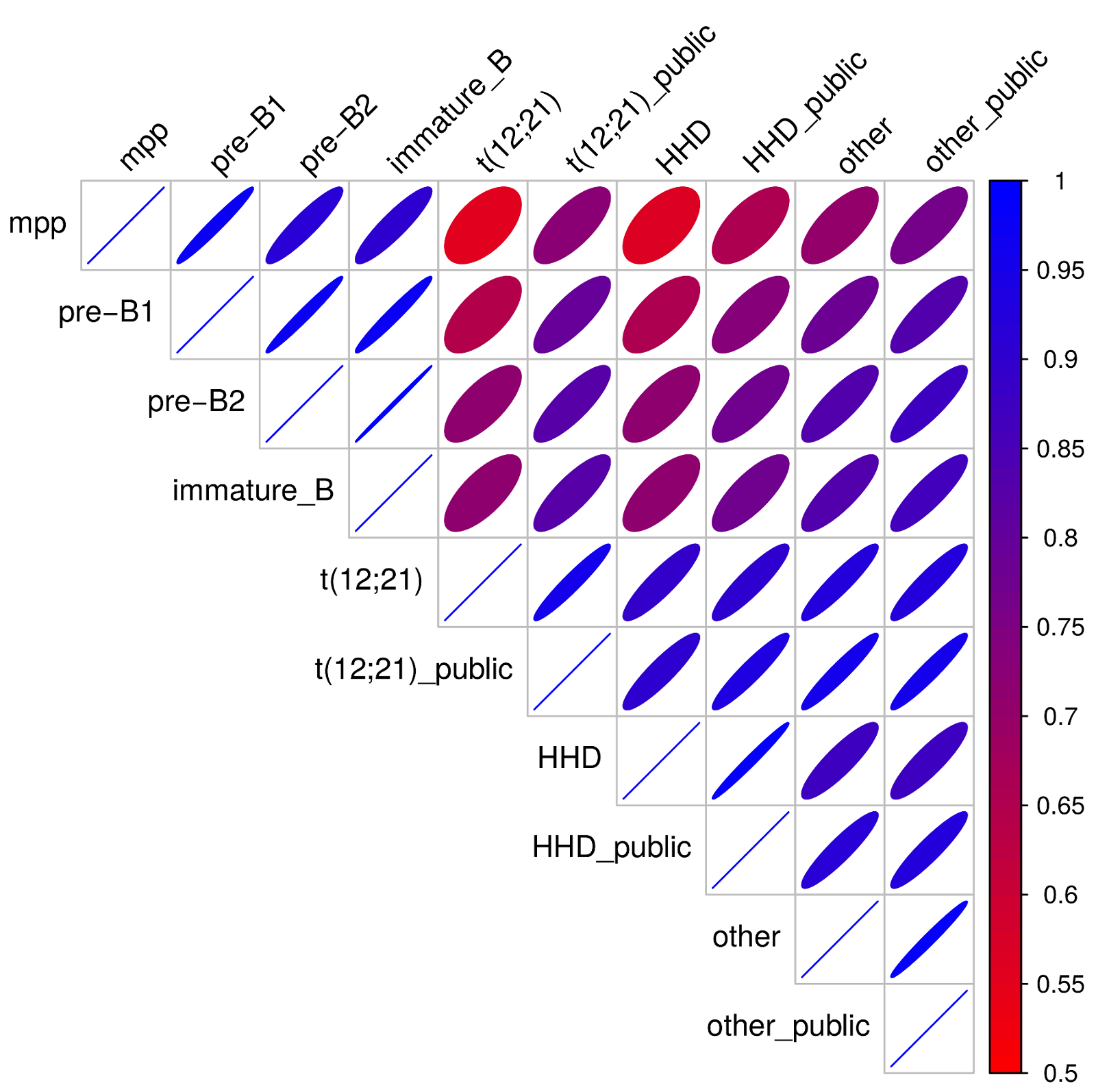

Supplement: S4 Fig — Pairwise Pearson correlations of 450K methylation levels of t(12;21) active promoters (n = 34) in four healthy B cell stages (mpp, preB-I, preB-II, immature B) and three cALL subtypes (t(12;21), HHD, ’Other’) from both in-house and public datasets (242 samples total). 450K beta values were used as methylation levels. For each promoter, methylation levels were obtained by averaging the values of all overlapping 450K probes. For more than half of t(12;21) active promoters (57%; 45 / 79), no probes overlapped. Circle size increases as Pearson correlation decreases. (TIF) [file pone.0207250.s004.tif]

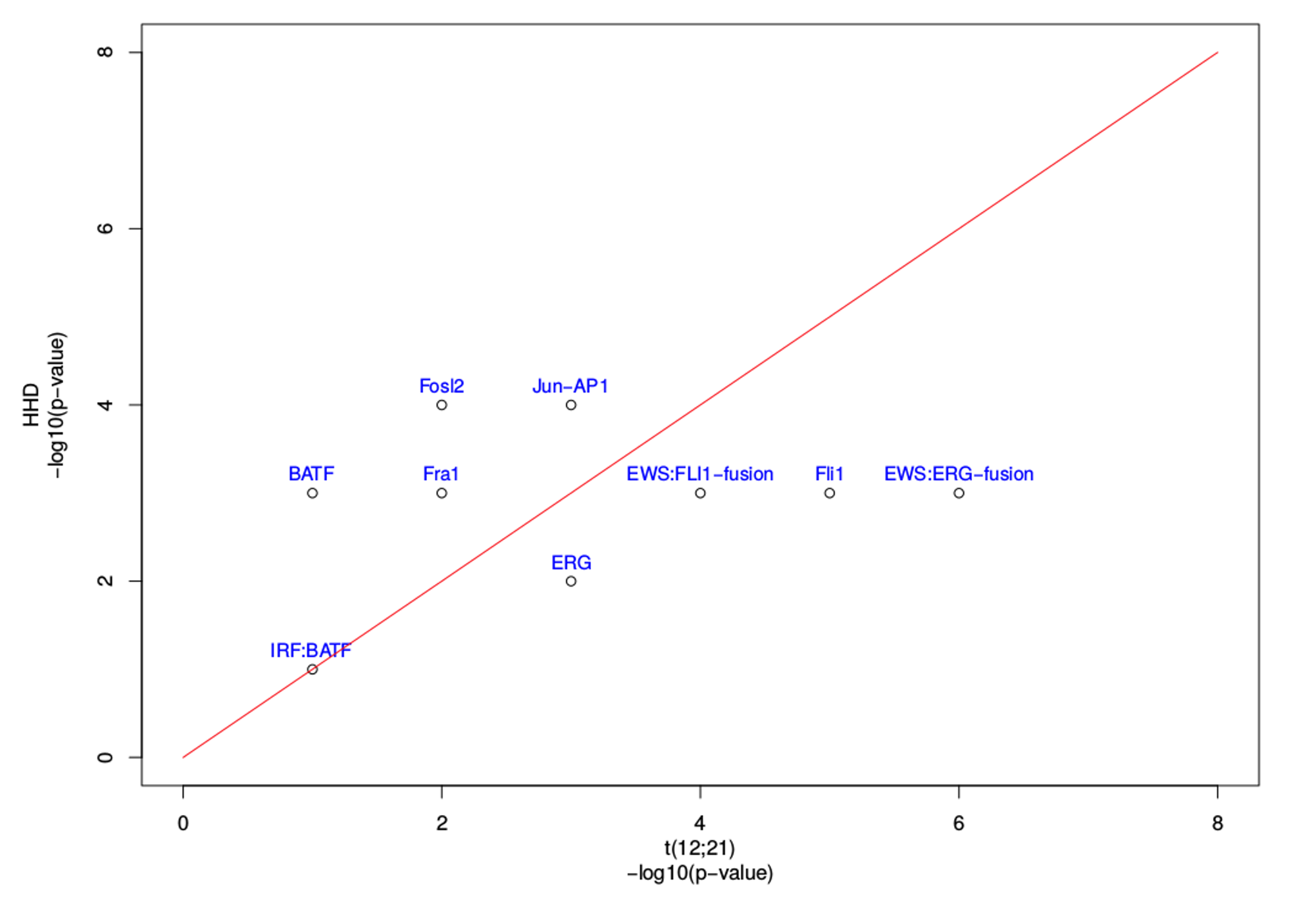

Supplement: S5 Fig — Significantly enriched motifs (q-value < 0.05) in candidate promoters (±10 kb around the 5’ start of vlincRNAs) of t(12;21) and HHD high expressed quartiles vlincRNAs (Q4, n = 64). (TIF) [file pone.0207250.s005.tif]
